# Development of infrared desolvation device for single cell element analysis using droplet sample injection inductively coupled plasma spectrometry

**DOI:** 10.1007/s44211-026-00888-z

**Published:** 2026-03-17

**Authors:** Kai Fukuchi, Syu Yamaji, Yuya Shimizu, Takashi Ohta, Akane Yaida, Yuki Maemoto, Motohide Aoki, Tomonari Umemura, Akitoshi Okino

**Affiliations:** 1 FIRST, Institute of Science Tokyo, Yokohama, 226-8501 Japan; 2 School of Life Sciences, Tokyo University of Pharmacy and Life Sciences, Hachioji, 192-0392 Japan

**Keywords:** Single cell analysis, Mass spectrometry, Desolvation, Elemental analysis

## Abstract

Microdroplet injection systems, which introduce single cells into an inductively coupled plasma by encapsulating them in droplets, have been developed by several research groups, including our group. In our laboratory, to improve the analytical sensitivity, a droplet desolvation system using a ribbon heater has achieved an approximately tenfold improvement in sensitivity. However, the desolvation throughput was limited to 100 Hz because of the low heat-transfer efficiency associated with indirect of indirect heating via heated gas. In this paper, a novel desolvation device using infrared radiation was developed to improve the desolvation throughput. Infrared radiation enables direct and efficient droplet heating. The developed device comprises a heating section and a cooling section. An infrared lamp with a luminous length of 140 mm was used in the heating section. Infrared radiation was focused on the flight axis of the droplets to enhance irradiation efficiency. The cooling section was placed downstream of the heating section to condense and remove vapor. With a power of 1000 W applied to the lamp and a carrier gas flow rate of 0.1 L min^−1^, the carrier gas temperatures at the bottom of the heating section remained below 71 °C for both argon and helium. Under these conditions, the cells remained intact without rupture. Helium exhibited superior throughput compared to argon and when the carrier gas flow rate was above 0.5 L min^−1^, the desolvation at a throughput of 1000 Hz could be achieved with an applied power of 150 W to the lamp.

**Graphical abstract:**

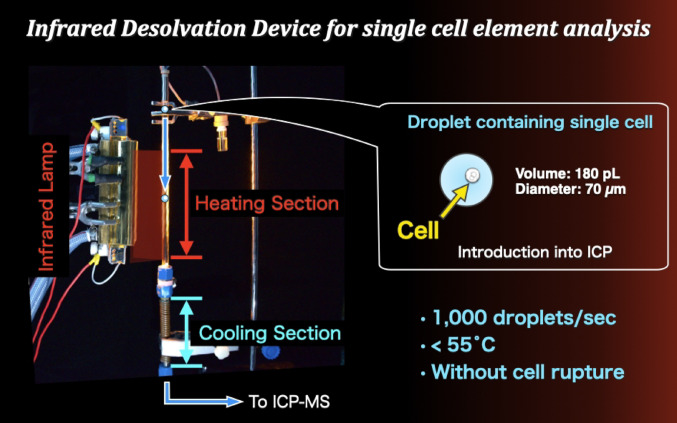

## Introduction

In the life science field, single cell analysis that focuses on cell-to-cell heterogeneity has attracted increasing attention in recent years [1–3]. In particular, applications targeting genes and proteins have advanced rapidly in areas such as drug discovery [4–6] and multi-omics, combining single cell analysis of different omics research [7–9]. In the field of single cell elemental analysis, metallomics, first proposed by Haraguchi in 2004 [10], aims to elucidate the roles of metals in biological systems. Despite extensive studies on cellular metal heterogeneity [11, 12], metal-based drug response [13], and nanoparticle uptake [14], the primary goal of metallomics, the analysis of all elements within a single human cell, has yet to be achieved, although nearly 20 years have passed since its proposed. This limitation primarily arises from the lack of analytical techniques capable of introducing intact single cells into multi element detectors with sufficient sensitivity and throughput.

To better understand the analytical framework of this study, it is important to briefly describe the principles and detection limits of inductively coupled plasma (ICP)-based analytical techniques. As techniques for trace element analysis, ICP optical emission spectroscopy (ICP-OES) and ICP mass spectrometry (ICP-MS) are widely used. These methods use high-temperature plasma as an excitation source or ionization source. For many elements, the typical detection limits of ICP-OES range from sub-ppb to several ppb (approximately 0.1–10 µg L^−1^), whereas those of ICP-MS extend down to the ppt–sub-ppb range (approximately 0.1–10 ng L^−1^), depending on the element, matrix, and introduction system. To analyze elements using these methods, liquid analytical samples are introduced into plasma by nebulization [15, 16]. Therefore, cells should be digested by acid before analysis to analyze elements within cells. Thus, while this method allows high-sensitivity analysis of the average information from many cells, it cannot analyze individual cells. To analyze intact cells, methods for single cell analysis without cell digestion, such as introducing cell suspension samples via nebulization, have been studied. Using this technique, Tanaka et al. reported results from analyzing yeast cells, unicellular algae, and red blood cells (RBCs) [17]. Additionally, Rodríguez et al. reported results from single cell analysis of the A2780 cell line using a self-made nebulizer [18]. The main drawback of the nebulization methods was low transport efficiency, but various innovations such as the shape of the spray chamber and gas flow have led to improvements [19–21]. Despite recent advancements, several critical challenges persist, including the simultaneous introduction of aggregated cells and physical cell damage during nebulization.

To address these challenges, droplet-based single cell introduction methods have been developed [22–25]. Encapsulation of single cells within droplets reduces mechanical stress during transport and enables precise control of cell introduction frequency, thereby enabling determination of the absolute detection efficiencies [26–28]. In our previous study, a microdroplet sample introduction system (M-DIS) was developed in which single cells are encapsulated within droplets approximately 70 µm in diameter and sequentially introduced into the ICP [28–32]. In M-DIS, droplets are ejected by applying a pulsed voltage to a piezoelectric actuator positioned around a glass capillary of the microdroplet generator (µDG). Encapsulating a single cell within this droplet enables the introduction of single cells into the ICP one by one. To further enhance analytical sensitivity, a droplet desolvation device was integrated upstream of the ICP to remove the solvent before introduction [30, 31]. This approach can prevent the analysis sample from passing through the plasma while still containing the droplet’s residual water and insufficient ionization. It also prevents reduction in plasma temperature due to droplet water vaporization, that reducing the analytical load [33, 34]. This device heats the gas inside a 300 mm long metal tube using ribbon heaters placed around it, to evaporate the water in the droplet. Subsequently, the vapor was condensed and removed in a cooling section [30, 31]. This improved the analytical sensitivity by approximately tenfold [31]. However, several technical issues remained due to the heating method. To achieve high throughput desolvation using conventional methods, the carrier gas must be heated to high temperatures, which can cause cell rupture. When cells rupture, their fragments disperse and are introduced into the ICP, causing cell-derived signals to spread temporally and decrease in intensity. Furthermore, because this desolvation device could desolvate only up to approximately 100 droplets s^−1^ and the sample had to be prepared at a concentration of less than one cell per 40 droplets to prevent more than one cell from being contained within a single droplet, the throughput for cell analysis was low, at 2.5 cells s^−1^ or less [31]. To achieve comprehensive single-cell elemental analysis, high throughput and highly sensitive analytical techniques are essential. For this purpose, an alternative heating approach which enables efficient solvent removal without cell rupture was required.

Therefore, in this study, a novel desolvation device was developed to improve desolvation throughput at low carrier gas temperatures. Previous reports indicate that thermal cell lysis occurs typically at 90–100 °C [35–38]. To avoid any potential impact on cell structure during the desolvation process, the threshold for the carrier gas temperature in this study was set at 80 °C. This ensures a safe margin below the reported lysis temperature, thereby maintaining the structural integrity of the cells. In this device, an infrared radiation was utilized as the heating source because water exhibits strong infrared absorption, direct radiative heating [39–41] enables efficient desolvation without excessive gas heating. Although the primary focus of this study is the development and performance evaluation of the IR desolvation device, replacing conventional heating units with this system is expected to realize high-sensitivity and high-throughput analysis. Theoretically, complete desolvation can enhance analytical sensitivity [30, 31], and this study provides the necessary technological basis to achieve this benefit at high throughput. This study focused on developing an infrared desolvation device and investigating the species and flow rate of the droplet carrier gas suitable for rapidly removing moisture from droplets, as well as the power applied to the infrared lamp.

## Materials and methods

### Cell culture

In the experiments, HeLa cells (derived from human cervical cancer cells) were obtained from RIKEN BRC (Tsukuba, Ibaraki, Japan) and cultured in Dulbecco’s Modified Eagle’s Medium (D-MEM; FUJIFILM Wako Pure Chemical Co., Osaka, Japan) supplemented with 10% fetal bovine serum (FBS; Peak Serum, Inc., Colorado, USA). Cells were seeded in 10 mL of the culture medium in a 10 cm diameter culture dish. Cultures were maintained in a humidified incubator at 37 °C with 5% CO_2_, and the medium was replaced every 2–3 days.

For cell harvesting, the culture medium was first removed by aspiration. The cells were then gently washed by slowly adding sterilized phosphate-buffered saline (PBS) along the dish wall to remove residual medium and debris. After aspirating the wash solution, 1 mL of sterile 0.05% trypsin–0.8 mmol/L EDTA solution was added, and the dish was incubated at 37 °C until the cells became detached and rounded, as confirmed by phase-contrast microscopy. To deactivate the trypsin, 10 mL of D-MEM supplemented with 10% FBS was subsequently added, followed by gentle pipetting to generate a single cell suspension. Finally, the cell suspension was transferred to a polypropylene centrifuge tube and kept at 4 °C until further processing.

### Sample preparation

The HeLa cell suspension was centrifuged at 1040×*g* for 1 min using a centrifuge (CN-810, AS ONE CORPORATION, Osaka, Japan). The supernatant was discarded, and the cells were resuspended in PBS. After repeating this procedure twice, the cell concentration was adjusted to 3.0 × 10^5^ cells mL^−1^, corresponding to one cell per 20 droplets. This concentration ensured that only one cell was present in each droplet. PBS and iodixanol solution (OptiPrep™, Serumwerk Bernburg AG, Bernburg, Germany) were used for sample preparation. Iodixanol solution was added at 10% (w/w) to prevent sedimentation and clogging during droplet ejection.

### Desolvation system

**Fig. 1:**
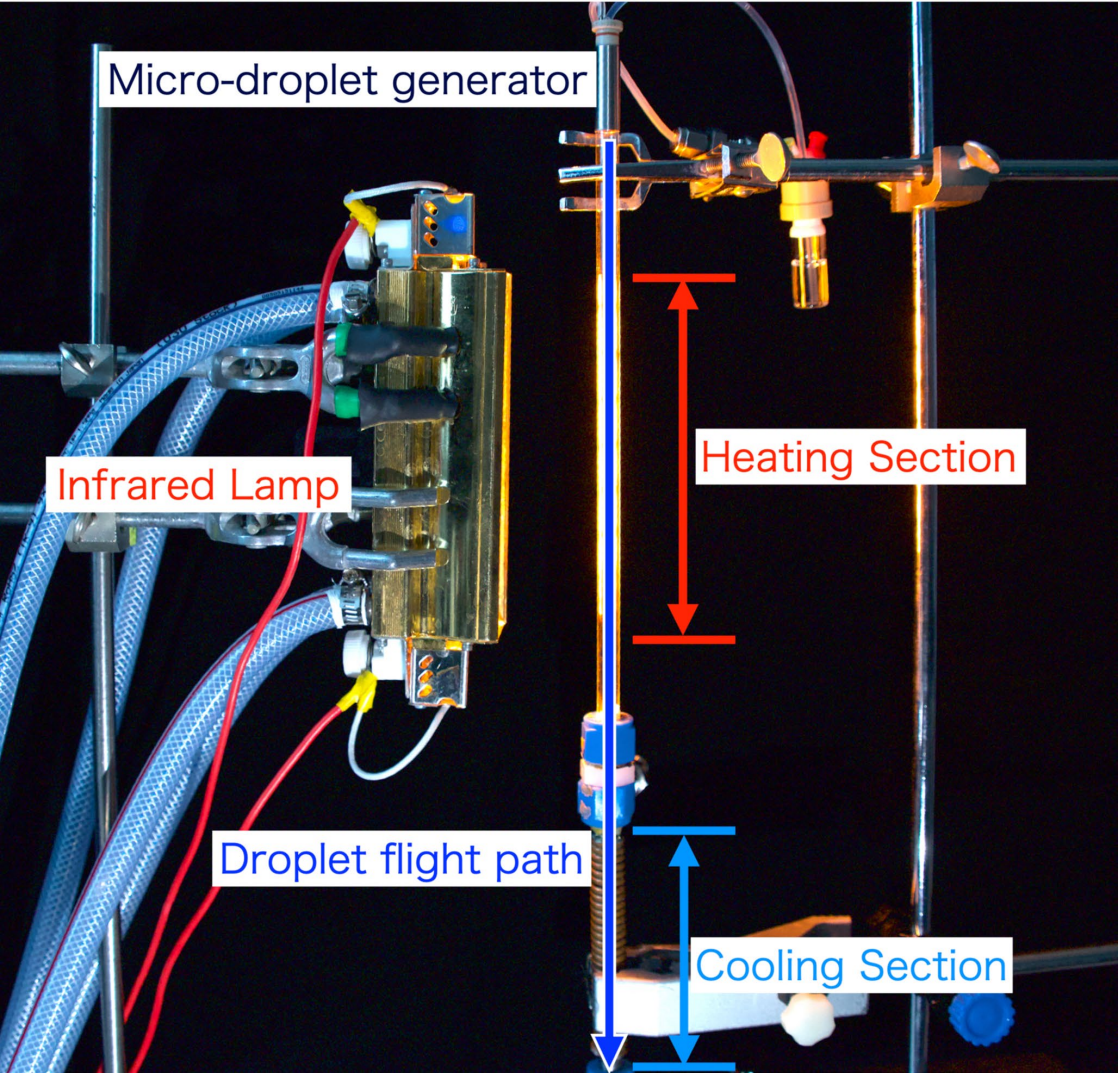
Photograph of the infrared desolvation device for the microdroplet injection system

**Fig. 2:**
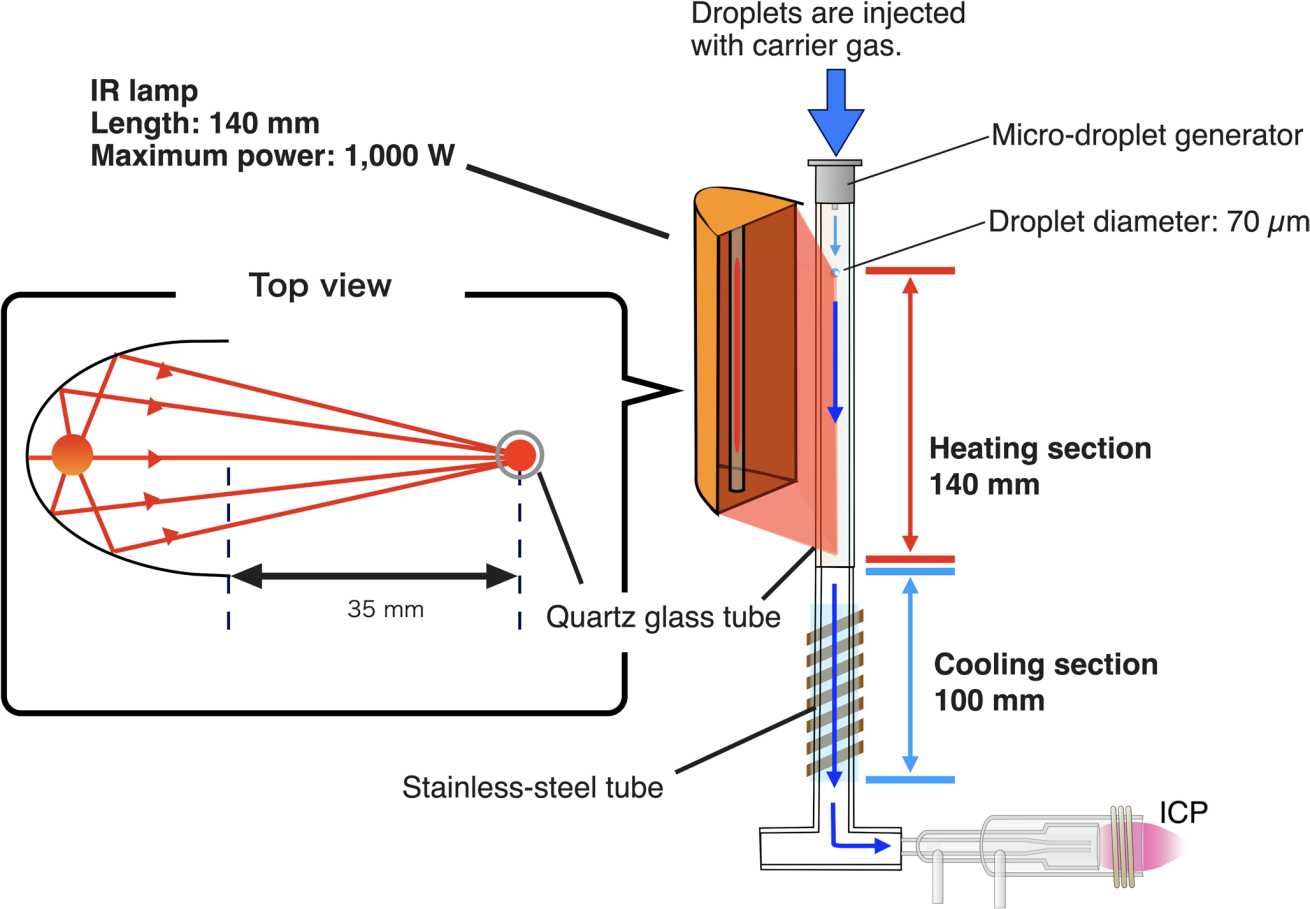
Schematic diagram of the infrared desolvation device developed for a single-cell elemental ICP-MS analysis system. The system includes a heating section irradiated by an infrared lamp and a cooling section for removing condensed solvent vapor

Figures 1 and 2 show a photograph and a schematic diagram of the developed infrared desolvation device. The desolvation device comprise a heating section and a cooling section. An infrared lamp (Light Warmer KSC140-35, K Sonic Co., Ltd., Saitama, Japan) with an emission length of 140 mm and a maximum output of 1000 W was used as the radiation source. The main range of the wavelength irradiated from this lamp is approximately 0.7–1.6 µm and the peak is at approximately 1 µm. It employs a reflective focusing optical system to concentrate the light at a distance of 3.5 mm. A quartz glass tube with an outer diameter of 9.0 mm and an inner diameter of 7.0 mm was used for the droplet transport path. Quartz glass has high infrared transmittance (approximately 99% transmission at 1 mm thickness), enabling low-loss infrared radiation [42]. The quartz glass tube was positioned such that the infrared focusing axis was aligned with the central axis of the quartz glass tube. The cooling section was fabricated by wrapping a copper tube (o.d. 3 mm, i.d. 2 mm) around a stainless steel tube (o.d. 9.5 mm, i.d. 9 mm). The cooling section length was 100 mm. Ethanol cooled to − 20 °C was circulated through the copper tube to cool the gas inside the stainless steel tube.

Droplets were introduced with the carrier gas from the top of the quartz glass tube. The droplets were irradiated with infrared light and heated while passing through the heating section. Afterward, the droplets and carrier gas then entered the cooling section, where the vaporized solvent was condensed and trapped as ice on the cooling section transport tube surface. As reported previously [30, 31], moisture removal from the carrier gas decreases the effects of vapor on analytical sensitivity. Regarding transport efficiency, this ice does not affect the process because the amount of deposited ice would be 0.3 mm thick even if droplets were introduced at a frequency of 1000 Hz. Argon or helium, both inert noble gases, were used as the carrier gases for droplet transport to suppress the formation of polyatomic ions and minimize spectral interferences in ICP-MS analysis.

### Measurement of gas temperature

The carrier gas temperature in the heating section was measured to confirm that is below 80 °C. The gas flow rate was maintained at 0.1 L min^−1^. A thermocouple (Ad-1214, A&D Company, Limited, Tokyo, Japan) was used for measurement, taking readings 10 mm from the lower end of the heating section. The power applied to the infrared lamp was set to 1000 W, and the temperature was recorded every 5 s for approximately 10 min. The measurements were repeated three times. The average temperature of each measurement was calculated from the data obtained between 450 and 600 s. The averaged temperatures were used to evaluate the average and standard deviation of each gas species. After 10 min of heating, the temperature of the quartz glass tube was measured using a thermographic camera (3-634-01, AS ONE CORPORATION, Osaka, Japan). Droplets were not introduced during the measurements. If these droplets had been ejected, the adhesion of droplets on the thermocouple made it difficult to measure the gas temperature. However, when droplets are introduced, the temperature will not exceed the measured value due to evaporative cooling; thus, this is considered to have no significant impact on the results.

### Desolvation effect evaluation method

**Fig. 3:**
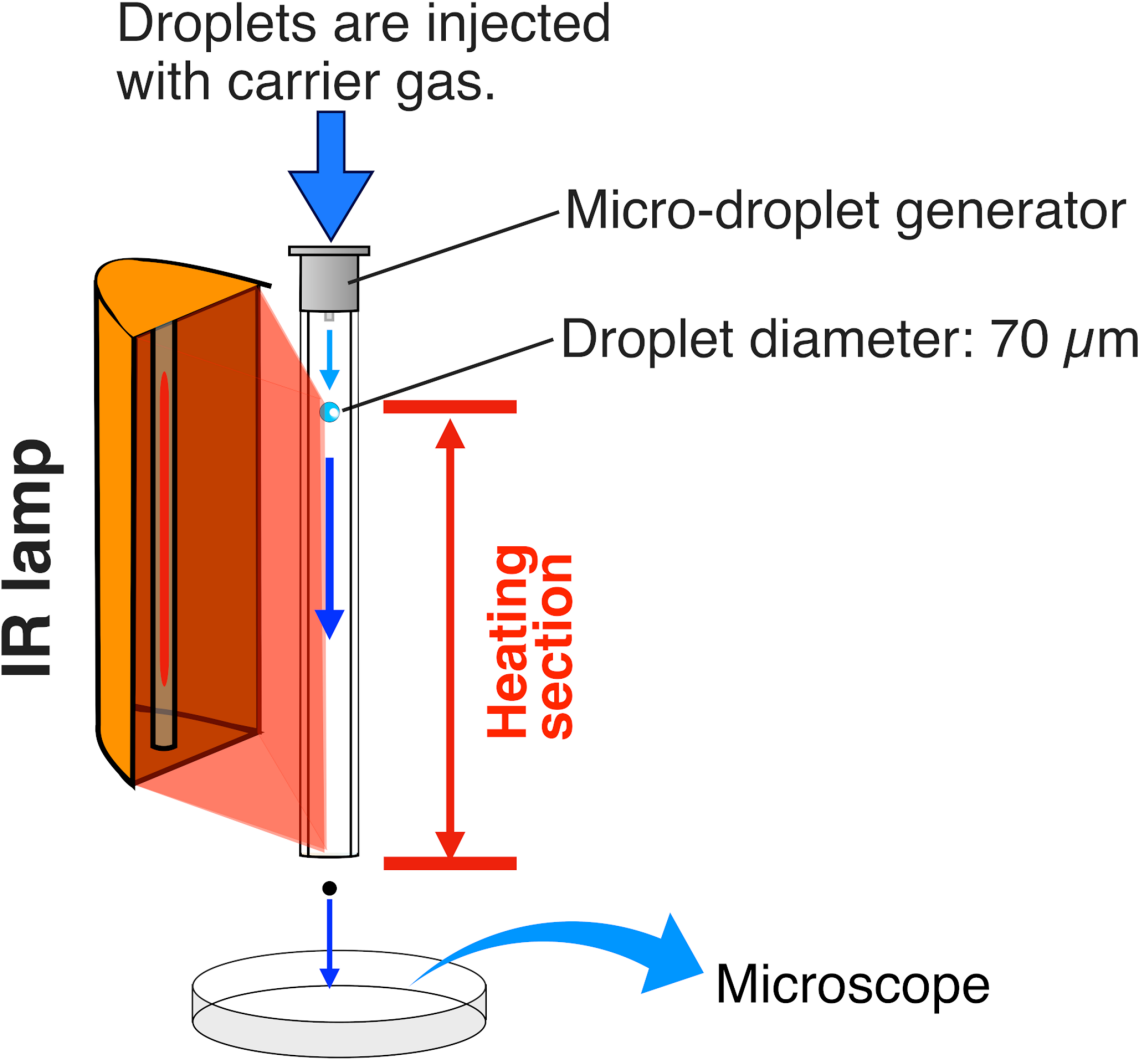
Experimental setup for evaluating the desolvation effect using the infrared desolvation device

The desolvation effect was evaluated, as shown in Fig. 3. Droplets that passed through the heating section were observed under a microscope (BZ-X710, KEYENCE, Osaka, Japan), and their diameters were measured for evaluation. In the experiment, a µDG (MD-K-150, microdrop Technologies GmbH, Norderstedt, Germany), previously developed for our droplet introduction method [28–32], was used to eject cell suspension droplets. Droplets were introduced into the desolvation device from the top of the quartz glass tube with the carrier gas. The µDG ejects droplets by applying a pulse voltage to a piezoelectric actuator arranged around a glass capillary. The voltage magnitude and pulse width applied to the piezoelectric actuator were adjusted for each experiment to ensure that the droplets did not fragment and flew vertically.

The power applied to the infrared lamp was set to 1000 W to investigate the carrier gas conditions in the desolvation device. The droplet ejection frequency was varied from 200 to 1000 Hz in 200 Hz increments. Argon or helium was used as the carrier gas, with flow rates of 0.1, 0.5, and 1.0 L min^−1^ for the experiments. Furthermore, to investigate the applied power enabling high throughput and non-destructive desolvation that prevents cell rupture, helium was used as the carrier gas with flow rates set to 0.5 L min^−1^ or 1.0 L min^−1^, while operating at a droplet frequency of 1000 Hz.

## Results and discussion

### Carrier gas temperature

**Fig. 4:**
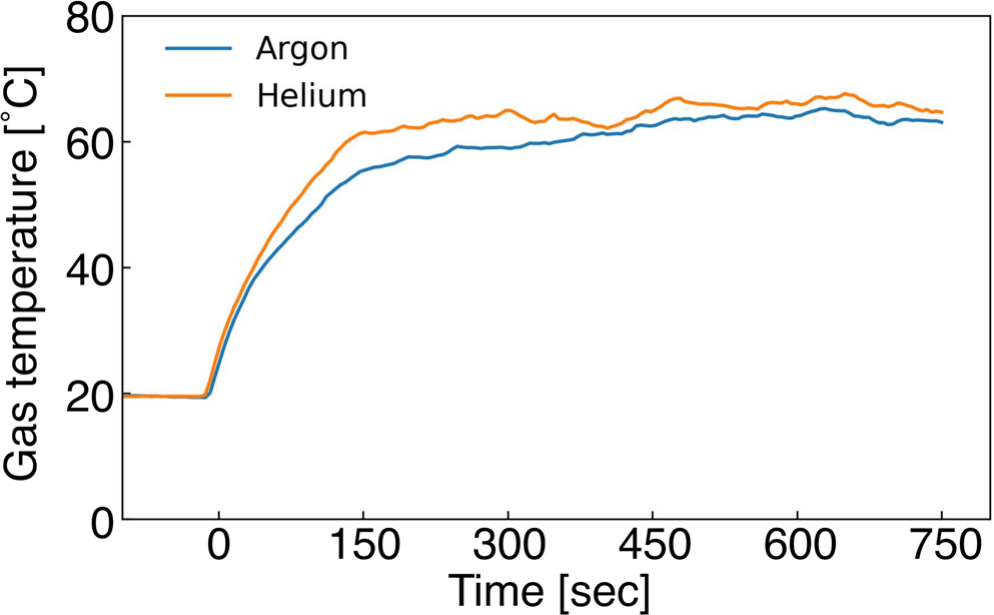
Temporal change in carrier-gas temperature under IR-lamp heating at 1000 W for argon and helium flows

Figure 4 shows the measured carrier gas temperature. The start time of the infrared heating was set to 0 s. The average temperature of each gas species was 67.0 ± 4.0 °C for argon and 70.3 ± 3.2 °C for helium. The average of the highest temperatures on the surface of the quartz glass tube was 132 ± 9.6 °C for argon and 135 ± 5.5 °C for helium. Since noble gases absorb almost no infrared radiation, gas heating was thought to occur via heat conduction from the quartz glass tube heated by infrared radiation. The molar specific heats of argon and helium do not differ significantly. Therefore, the difference in the gas temperature is thought to have arisen from the measurement deviation. These temperatures were sufficiently below 80 °C indicating that cell rupture due to excessive thermal stress can be avoided.

### Effects of carrier gas conditions

**Fig. 5:**
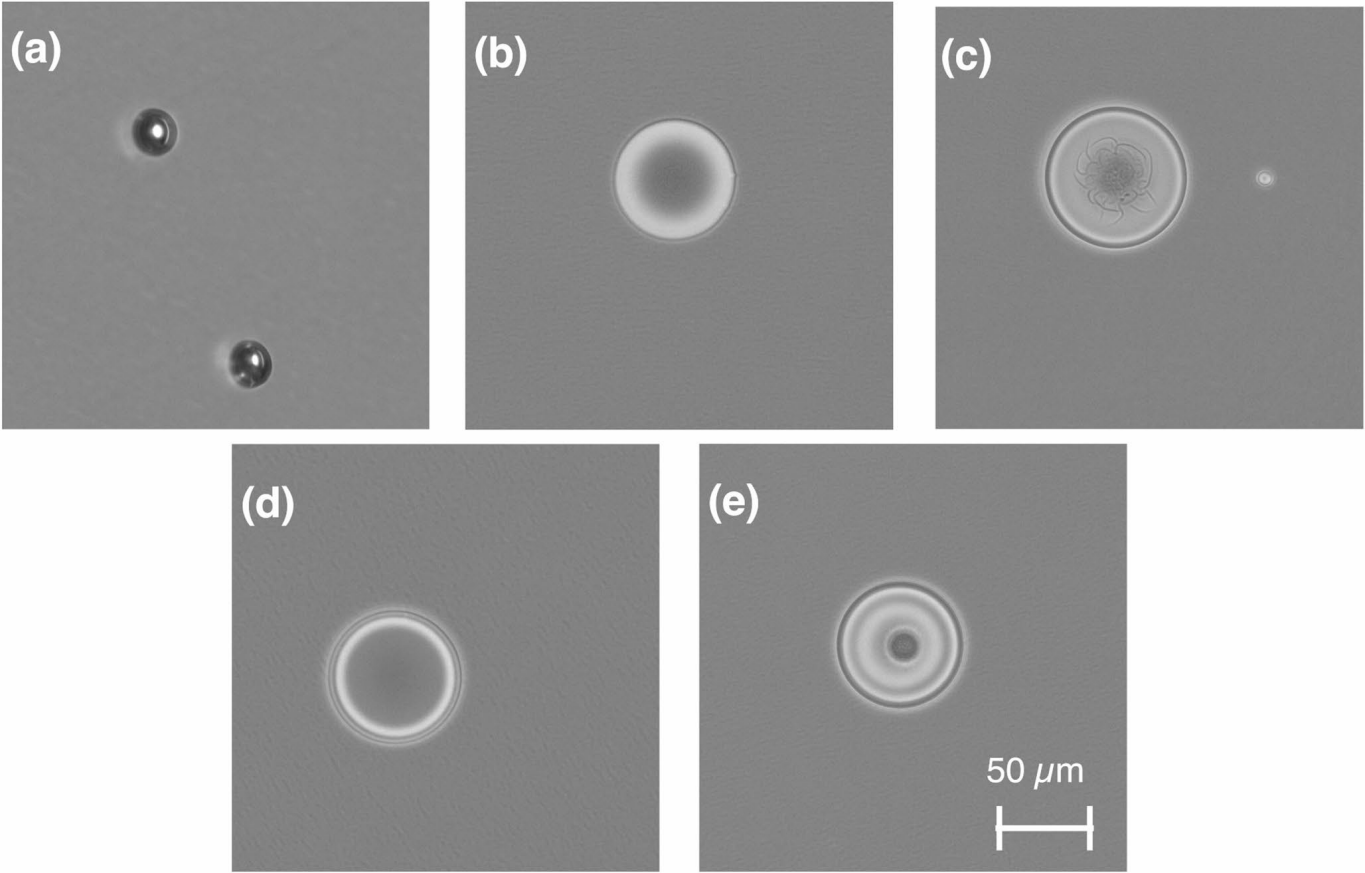
Phase-contrast micrographs of cell suspension droplets after passing through the heating section under various injection frequencies (argon, 0.1 L min^−1^). **a**–**e** Droplet appearance at 200, 400, 600, 800, and 1000 Hz, respectively. Droplets showing high-contrast granular textures correspond to fully desolvated particles

Figure 5 shows the microscope images of the cell suspension droplets desolvated using argon as the carrier gas at a flow rate of 0.1 L min^−1^. At 200 Hz, high-contrast granular particles were observed under phase-contrast microscopy. The diameters of these particles were consistent with those of the cells. If the solvent salts from droplets without cells had aggregated into single crystals, their diameter would be approximately 10 µm. Thus, the observed particles are likely composed of cells surrounded by salts. The absence of aggregated salt particles from cell-free droplets may be attributed to the rapid and simultaneous heating of the entire droplet by infrared radiation. Under these conditions, the salts may not have aggregated into particles large enough to be observed by microscopy. However, further investigation is required to confirm whether each particle contains a cell and to assess the cellular conditions. The cells without rupture encapsulated by salts that crystallized after solvent evaporation. At frequencies above 400 Hz, no such granular contrast was observed, and droplets with residual water were observed. Desolvation was defined as complete when the mean droplet diameter no longer significantly decreased with increasing irradiation power or injection frequency, indicating that further solvent removal had reached a limit. In the phase-contrast images, droplets showing strong optical contrast were used only as visual cues for dryness, whereas the quantitative criterion for complete desolvation was based on the statistical convergence of the mean droplet diameter—that is, when the mean value remained constant within one standard deviation.

**Fig. 6:**
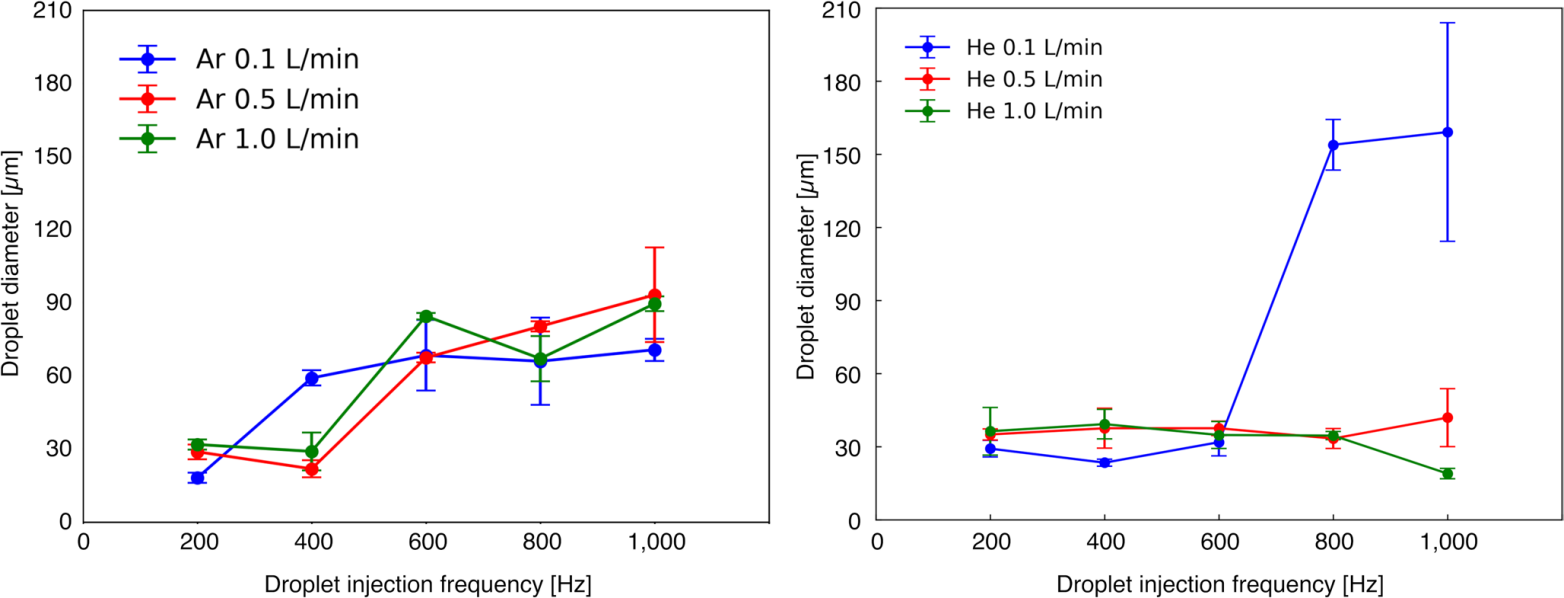
Effect of droplet injection frequency and carrier-gas flow rate on droplet diameter. **a** The results of argon. **b** The results of helium

The measured diameters are plotted in Fig. 6. In Fig. 6, the decrease in droplet diameter represents the desolvation progress because solvent evaporation reduces the droplet volume. For argon, the droplet diameter increased above 400 Hz. This behavior is likely due to by incomplete evaporation and partial coalescence between successive droplets. In contrast, for helium, the droplet diameter continuously decreased with increasing injection frequency, indicating efficient evaporation. At high ejection frequencies, the preceding droplet may not be fully evaporated before the subsequent one enters the heating zone, resulting in droplet merging or residual moisture. Consequently, when the carrier gas flow rate was 0.1 L min^−1^, desolvation was achieved at 200 Hz for argon and up to 400 Hz for helium. Higher droplet injection frequencies result in shorter inter-droplet distances. The observed decrease in desolvation efficiency is attributed to subsequent droplets passing through areas with insufficient diffusion of evaporated moisture. At the flow rate of 0.1 L min^−1^ and frequencies above 800 Hz, the droplet diameter under the helium condition was larger than that under the argon condition. If reproducibility is observed for the difference between He 0.1 L min^−1^ and Ar 0.1 L min^−1^, but interpretation remains difficult.

Comparing the results for argon and helium, helium showed a higher desolvation effect. This is considered because helium has a larger mutual diffusion coefficient with water than argon with water. The diffusion coefficient is a constant that is related to the rate at which one substance diffuses into another when two different substances are in contact. When substances A and B are in contact, the diffusion coefficient D_AB_ can be calculated by the Eq. (1) [43–45].

1 $$\begin{array}{c}{D}_{AB}=\frac{0.001858{T}^\frac{3}{2}}{P{\sigma }_{AB}^{2}\Omega }\sqrt{\frac{1}{{M}_{A}}+\frac{1}{{M}_{B}}}\end{array}$$

Here, T, P, σ_AB_, Ω, and M represent the gas temperature, pressure, collision cross section, collision integral, molecular weight, respectively. Assuming that the pressure and gas temperature are equal,${\mathrm{D}}_{{{\mathrm{Ar}},{\mathrm{H}}_{2} {\mathrm{O}}}} /{\mathrm{D}}_{{{\mathrm{He}},{\mathrm{H}}_{2}{\mathrm{O}}}}$ is 0.28. The collision cross section and collision integral values from Hirschfelder et al. [46] were used. From the above calculations, since the diffusion coefficient of argon is smaller than that of helium, the solvent from the evaporated droplet diffused more slowly. In addition, while a decrease in gas temperature likely occurred due to evaporation, the higher thermal conductivity of helium is thought to have mitigated this effect, thereby causing helium to exhibit a higher desolvation effect than argon.

Furthermore, when the carrier gas flow rate was set to 0.5 L min^−1^ or 1.0 L min^−1^ a higher desolvation effect was obtained than when the flow rate was set to 0.1 L min^−1^. This demonstrated that desolvation was possible up to 400 Hz when argon was used and up to 1000 Hz when helium was used. Increasing the carrier gas flow rate lowers the gas pressure, thereby increasing the diffusion coefficient. This is thought to have improved the desolvation effect. The above results indicated that the desolvation device developed in this study may improve the analytical throughput of single cell analysis using the droplet sample introduction method.

### Investigation of the IR lamp power condition

**Fig. 7:**
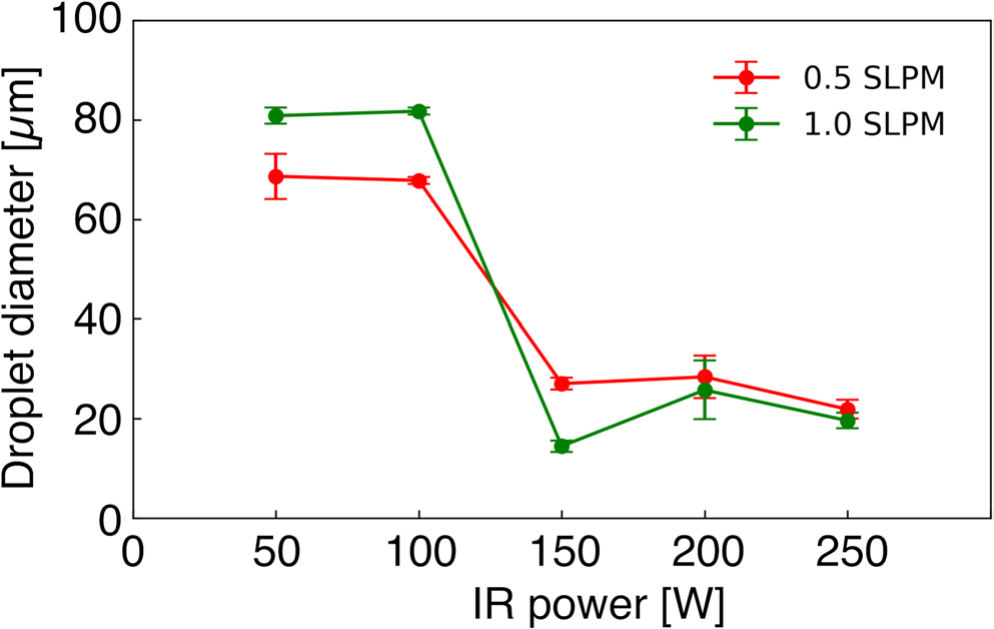
Relationship between IR-lamp power and droplet diameter for determining the minimum power required for complete desolvation

The results of desolvation performed by varying the power applied to the infrared lamp are shown in Fig. 7. Experiments were conducted by varying the power applied to the infrared lamp in 50 increments from 50 to 250 W. The results indicate that desolvation was possible at 150 W or higher for both flow rates of 0.5 and 1.0 L min^−1^. These results demonstrate that IR radiation is the dominant mechanism for evaporation in this desolvation device Regarding energy, a total of 0.44 mJ is required to completely vaporize the water in a droplet. The droplet was ejected at 2 m s^−1^ and introduced into the quartz glass tube. The viscosity of helium was 1.99 × 10^−5^ (Cited from NIST Technical Note 1334). Based on these values, the droplets passed through the heating section with the carrier gas over approximately 211 ms. The energy absorbed by the droplet as it passes through the heating section is estimated to be approximately 80 mJ when 150 W was applied to the infrared lamp. This calculation assumes infrared light is focused to a filament thickness of 1.5 mm, an infrared transmittance of 0.1 cm^−1^ for quartz glass, and an infrared absorption coefficient of 0.36 cm^−1^ for water at the wavelength of 1 µm [42]. Although theoretical calculations show that lower power should be sufficient, practical limitations, such as imperfect optical focusing and nonradiative energy losses necessitated higher applied power.

This desolvation device enables the gentle removal of moisture from droplets before introducing the sample into the plasma, thereby preventing the sample from passing through the plasma while retaining moisture. Even with pneumatic nebulizers commonly used in ICP-MS, incomplete desolvation occurs in the plasma when droplet diameters are approximately 15 µm or larger, leading to reduced analytical sensitivity [33, 34]. In this study, introducing droplets with a diameter of approximately 70 µm into the plasma requires approximately 100 times more energy to vaporize the water than for droplets of 15 µm diameter. Therefore, an increase in the frequency of incomplete desolvation is anticipated. The infrared desolvation device developed in this study is expected to enable high-throughput single cell elemental analysis with high analytical sensitivity by rapidly removing moisture prior to sample introduction.

## Conclusion

An infrared desolvation device was developed to improve the throughput and stability of single-cell elemental analysis using droplet sample introduction. The system achieves efficient solvent removal through infrared radiation heating, allowing low-temperature desolvation (≤ 70 °C) while preventing cell rupture. Using helium as the carrier gas and optimizing the infrared lamp power (≥ 150 W) enabled a throughput of 1000 Hz, which is markedly higher than that obtained with conventional systems. However, further investigations are required to evaluate the cellular conditions and the exact ratio of cell-encapsulating particles among the desolvated droplets. This approach offers a practical route for integrating nondestructive, high-throughput droplet desolvation with ICP-MS, potentially enabling quantitative single-cell elemental analysis at rates exceeding 50 cells s^−1^.

## Data Availability

The datasets generated and analyzed during the current study are available from the corresponding author upon reasonable request.
